# Intestinal Upregulation of Melanin-Concentrating Hormone in TNBS-Induced Enterocolitis in Adult Zebrafish

**DOI:** 10.1371/journal.pone.0083194

**Published:** 2013-12-20

**Authors:** Brenda M. Geiger, Beatriz Gras-Miralles, Dimitrios C. Ziogas, Apostolos K. A. Karagiannis, Aileen Zhen, Paula Fraenkel, Efi Kokkotou

**Affiliations:** 1 Division of Gastroenterology, Beth Israel Deaconess Medical Center, Harvard Medical School, Boston, Massachusetts, United States of America; 2 Hematology/Oncology, Beth Israel Deaconess Medical Center, Harvard Medical School, Boston, Massachusetts, United States of America; University of South Carolina School of Medicine, United States of America

## Abstract

**Background:**

Melanin-concentrating hormone (MCH), an evolutionarily conserved appetite-regulating neuropeptide, has been recently implicated in the pathogenesis of inflammatory bowel disease (IBD). Expression of MCH is upregulated in inflamed intestinal mucosa in humans with colitis and MCH-deficient mice treated with trinitrobenzene-sulfonic acid (TNBS) develop an attenuated form of colitis compared to wild type animals. Zebrafish have emerged as a new animal model of IBD, although the majority of the reported studies concern zebrafish larvae. Regulation MCH expression in the adult zebrafish intestine remains unknown.

**Methods:**

In the present study we induced enterocolitis in adult zebrafish by intrarectal administration of TNBS. Follow-up included survival analysis, histological assessment of changes in intestinal architecture, and assessment of intestinal infiltration by myeloperoxidase positive cells and cytokine transcript levels.

**Results:**

Treatment with TNBS dose-dependently reduced fish survival. This response required the presence of an intact microbiome, since fish pre-treated with vancomycin developed less severe enterocolitis. At 6 hours post-challenge, we detected a significant influx of myeloperoxidase positive cells in the intestine and upregulation of both proinflammatory and anti-inflammatory cytokines. Most importantly, and in analogy to human IBD and TNBS-induced mouse experimental colitis, we found increased intestinal expression of MCH and its receptor in TNBS-treated zebrafish.

**Conclusions:**

Taken together these findings not only establish a model of chemically-induced experimental enterocolitis in adult zebrafish, but point to effects of MCH in intestinal inflammation that are conserved across species.

## Introduction

Melanin-concentrating hormone (MCH) was initially purified from salmon pituitaries and as its name indicates, it induces fish skin pallor in response to environmental cues by segregating the pigment-containing granules in melanocytes [1]. Human and rodent melanin-concentrating hormone (MCH) is an identical 19-aminoacid neuropeptide primarily expressed in the lateral hypothalamus and zona incerta [2]. The gene for MCH, *pmch*, encodes for a prepropeptide, which is cleaved near the C-terminus to generate MCH and two additional neuropeptides, NEI and NGE, of unknown function. In mammals, MCH has a role in the central control of appetite that is supported by several genetic and pharmacological studies. For instance, MCH levels were found to be upregulated in the hypothalamus of leptin-deficient, *ob/ob* mice and fasting increased further its expression in both normal and obese mice [2]. Acute administration of MCH in the rat brain increases food intake while chronic administration results in excessive weight gain and insulin resistance [3]. Consistent with these observations, transgenic mice overexpressing MCH were hyperphagic and obese [4]; conversely, MCH-deficient mice exhibit a lean phenotype and are resistant to diet-induced obesity [5,6].

In zebrafish (*Danio rerio*) and other teleost genomes two distinct MCH genes, *pmch1* and *pmch2* were identified [7]. The mature peptides, MCH1 (17-aa) and MCH2 (19-aa), have 13 amino acids in common, but show higher homology with MCH from other species than between them. Specifically, MCH1 is identical to salmon MCH and MCH2 is only 3 amino acids different from human and mouse MCH. Based on sequence homology, genomic structure, synteny and functional analysis, MCH2 appears to be the zebrafish ortholog of the mammalian MCH [7]. Detailed mapping studies of MCH1 and MCH2 expression in zebrafish have focused primarily on the brain. By in situ hybridization analysis, in adult zebrafish, among other brain regions, transcripts for pmch1 and pmch2 were found in non-overlapping areas of the tuberal nucleus in the hypothalamus, which corresponds to the arcuate nucleus in mammals, one of the important centers for the homeostatic regulation of feeding behavior [7]. In further support of the notion that the zebrafish MCH2 is the equivalent of mammalian MCH, a two-fold increase in immunoreactivity against mammalian MCH was found in the hypothalamus of fasted zebrafish, as has been demonstrated in fasted mice [7]. 

In humans, two MCH receptors have been described, MCHR1 and MCHR2, which are G-protein coupled and share 38% homology [8,9]. In rodents, MCHR1 is 95% identical to the human sequence, but MCHR2 appears to be a nonfunctional pseudogene in this species. MCHR1 and MCHR2 orthologs in zebrafish have been described, with two orthologs for MCHR1 MCHR1a and MCHR1b, as a result of a late genome duplication event [10,11], but a single ortholog of MCHR2. Based on RT-PCR analysis in zebrafish embryos and adult tissue [10], MCHR1a was present only in embryos (day 4-7). Expression of MCHR1b was detected in day 2 embryos and throughout development. In adult zebrafish, MCHR1b expression was higher in the head, although lower levels were also detected in the adult torso. Expression of MCHR2 seems to parallel that of MCHR1b, but is stronger in the body compared to the zebrafish head. 

We have recently reported induction of MCH in the inflamed intestine of humans and mice pointing to additional physiological effects of MCH outside of the central nervous system [12]. Despite detailed anatomical studies of the expression of *pmch* genes and peptides in the zebrafish brain [7], information about MCH in peripheral tissues in zebrafish is lacking. Given the sequence and functional conservation of MCH across species, in the present study we examined whether MCH is expressed in the zebrafish intestine and its potential regulation during intestinal inflammation, as it has been demonstrated in mammals [12,13].

A few models of chemical-induced enterocolitis have been developed in zebrafish [14], which are analogous to mouse experimental colitis and recapitulate aspects of human inflammatory bowel disease (IBD) such as neutrophilic infiltration, microbiome-dependence and response to anti-inflammatory drugs [15]. These include soaking zebrafish larvae in TNBS [16,17] or DSS solution [15], and intrarectal administration of oxazolone in adult zebrafish [18]. A significant limitation of the larvae model is extraintestinal effects of the chemicals due to epidermal exposure which might alter the course of intestinal inflammation [15]. An additional concern is the lack of adaptive immune responses prior to 4-6 weeks of zebrafish development [19,20]. Finally, full development of gut folds and intestinal cell lineage specification (i.e. goblet cells, and enteroendocrine cells) is completed after 2 weeks post-fertilization [21-23]. For these reasons, we chose to study adult zebrafish. In order to make direct comparisons as they relate to regulation of MCH between the current and our previous studies in mice with TNBS-induced colitis, we developed a TNBS-induced model of experimental enterocolitis. After a validation process, we used this model to demonstrate upregulation of MCH in the inflamed intestine of adult zebrafish as we have previously shown in mice with experimental colitis and in patients with IBD [12]

## Materials and Methods

### Ethics Statement

The Beth Israel Deaconess Medical Center’s Institutional Animal Care and Use Committee has approved the described studies in zebrafish.

### Induction of TNBS- enterocolitis in adult zebrafish

Wild-type 3-6 month old male zebrafish were purchased from EkkWill Waterlife (Ruskin, FL, USA) and kept in the zebrafish facility for at least one month for equilibration prior to induction of colitis. Myeloperoxidase–GFP transgenic (Tg(mpx:EGFP) ) zebrafish were a gift of L. Zon, Boston’s Children Hospital. For each experiment, fish were weight-matched, placed in stand-alone fish tanks (5 fish per 500ml tank) and fasted for 18 hours prior to induction of colitis. The fish tank water was replaced on a daily basis. Fish used in the experiments weighed 0.2 - 0.6 g. 

For the induction of chemical colitis, anesthetized fish were placed on their backs under a stereomicroscope, pressing gently on the belly so that the rectum protruded slightly, as previously described [18]. Gel loading micropipette tips were placed approximately 1 mm into the rectum to administer the TNBS solution (1 ul per 0.1 g of body weight). TNBS (2,4,6-trinitro-benzene-sulfonic acid-Fluka) was dissolved in 30% ethanol and a concentration range of 40 mM-320 mM was tested in dose-response experiments, based on previous studies using zebrafish larvae soaked in TNBS-containing media [14,17]. Vehicle alone (30% ethanol) was administered intrarectally in control zebrafish. After recovery from anesthesia, fish were returned to their original tanks. 

### Survival and microbiome effects

Survival of fish was monitored for 96 h, at 6-12 h intervals. To test the effect of the microbiome on the severity of colitis, 10, 50 or 100 mg/L of vancomycin hydrochloride (Sigma) was added to the fish tank water 18 h prior to intrarectal administration of TNBS and for the following 48 h. A third group of fish received only vancomycin. 

### Assessment of enterocolitis in adult zebrafish

Zebrafish were sacrificed 6 hours after TNBS or vehicle injection and the gastrointestinal tube was dissected en block, fixed for 24 hours in 4% paraformaldehyde and then frozen in O.C.T. media (Sakura Finetek). Five-micron sections were stained with hematoxylin-eosin for the histological evaluation of inflammation under a Zeiss Axioimager M1 Microscope, and photomicrographs were taken at 20x and 63x magnifications.

Histological measurements were performed in 4 fish per group (TNBS or vehicle treatment) as follows: Sagittal sections from three different regions of the intestine from each fish were selected to represent similar areas among all fish based on anatomical landmarks and images were captured under the same magnification (10X objective). The images were printed in color and the villi height and thickness were measured using a ruler by the same blinded observer. For the abundance of goblet cells, a scoring system was used (0-5). For data analysis, the average values from each fish were used.

In another series of experiments, the brain and the gastrointestinal tube were removed from fish treated with TNBS or vehicle for 6 hours, and immediately frozen in liquid nitrogen. Total RNA was extracted using Trizol (Invitrogen) and purified using the RNeasy mini-kit (Qiagen). One microgram of RNA was reverse-transcribed into cDNA using the Advantage RT for PCR reagents with oligo(dT) (Clontech Inc). For each sample and gene, RT minus reactions were also included. Quantitative gene expression was assessed by real-time PCR using Sybr Green PCR Master Mix (Applied Biosystems) in an ABI PRISM 7700 Sequence Detection System. A list of gene-specific primers for zebrafish *IL-1beta*, *IL-8*, *IL-10*, TNFalpha, *pmch1*, *pmch2*, *MCHR1b*, *MCHR2* and *TBP* is provided in Table S1 [10,18,24-26]. Results are expressed relative to vehicle-treated control (control=100 arbitrary units (AU)), unless indicated otherwise). TBP was used as a housekeeping gene and its expression was similar between treated and untreated groups.

### Flow-cytometry analysis

We administered TNBS (160 mM) or vehicle intrarectally into Tg(mpx:EGFP) adult zebrafish and sacrificed the animals 6 hours later. The gastrointestinal tract was dissected en block and the tissue was teased through a 40um cell strainer (BD Falcon) in a 50ml conical tube using a syringe plunger and rinsed with 3 mL of PBS containing 2% heat-inactivated fetal bovine serum (FBS). The cell suspension was centrifuged for 10 minutes at 1000 rpm and 4°C. The supernatant was removed and cells were re-suspended in 200 ul of FACS buffer. Cells were analyzed by flow-cytometry (LSRII, BD) as described elsewhere [18]. Percentages of GFP-positive cells are presented in a graph.

### MCH Immunostaining

OCT preserved frozen sections (5 um) of intestinal tissue from fish treated with TNBS (160 mM) or vehicle for 6 hours, as described above, were washed in phosphate-buffered saline with 1% Triton-100 (PBS-T) and blocked with Protein Block Serum-Free (Dako). The fixed tissues were then incubated at room temperature for 2 hours with a rabbit primary antibody raised against the human/mouse MCH peptide (1:300), which has been characterized in detail in previous studies [12,27], followed by incubation with Alexa Fluor 594 Donkey anti-rabbit antibody (Molecular Probes, dilution 1:300) for 30 minutes at room temperature. Sections were then mounted with Prolong Gold 4’,6-diamidino-2-phenylindole (DAPI) mounting solution (Invitrogen) and visualized under a Zeiss LSM510 META Confocal System. In negative control sections, the primary antibody was omitted in the procedure. 

### Sequence comparisons

Amino acid homology of MCH and its receptors across species (*Homo sapiens, Mus musculus*, and *Danio rerio*) was investigated through multiple sequence alignments using ClustalW 2.1 Software. Identical residues have been highlighted. Accession numbers for MCH and its receptors are: *Homo sapiens* AAA63214.1 (pMCH), NP_005288.3 (MCHR1), NP_001035269.1 (MCHR2); *Mus musculus* EDL21464.1 (pMCH), NP_660114.1 (MCHR1); *Danio rerio* ACJ64087.1 (pMCH1), ACJ64086.1 (pMCH2), AAO24752.1 (MCHR1a), AAO24753.1 (MCHR1b), AAO24754.1(MCHR2).

### Statistical analysis

Data are shown as mean ± standard error. Statistical analysis was performed using StatView 5.0.1.Software. Mann-Whitney non-parametric test or t-test were used to evaluate differences between TNBS and vehicle-treated groups, unless indicated otherwise. Kaplan-Meier analysis, followed by log-rank test, or chi-square, was used in the survival experiments. The reported number of animals per experimental group is the actual number included in the statistical analysis. Animals that did not survive the first 30 minutes of the experiment due to problems with anesthesia or intestinal puncture during the intrarectal infusions were excluded from the study.

## Results

### TNBS-induced enterocolitis in adult zebrafish

**Figure 1:**
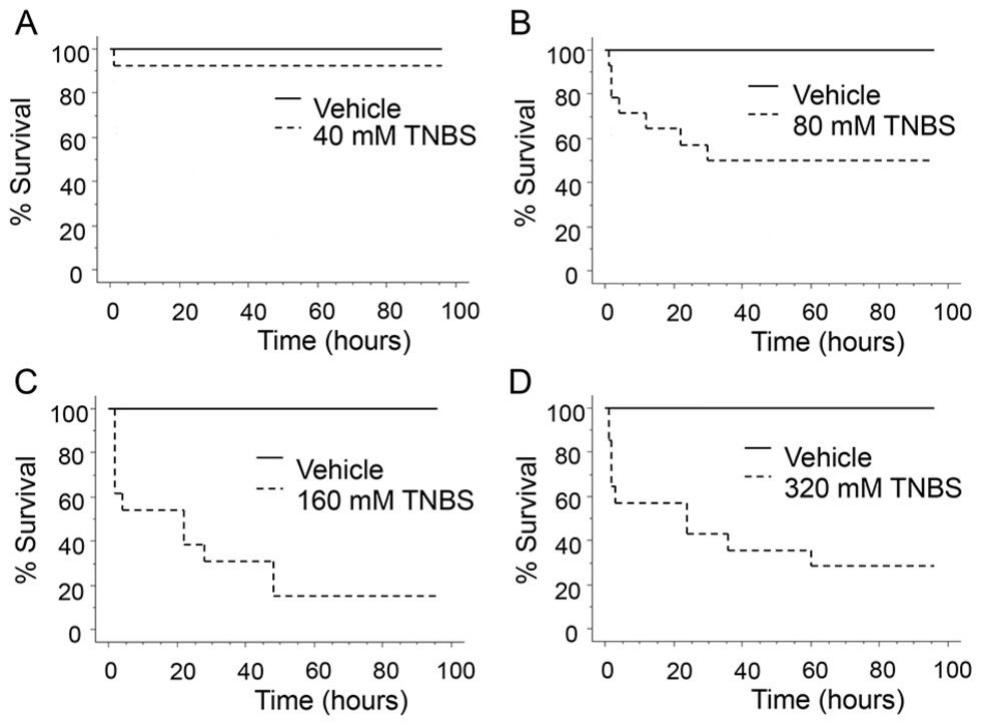
Adult zebrafish are susceptible to TNBS-induced enterocolitis. Kaplan-Meier survival analysis in response to different concentrations of TNBS (40mM-320mM). TNBS was administered by intrarectal infusion and doses were adjusted for body weight to a volume of 1 uL/0.1 g of body weight. Fish were monitored for a total of 96 hours in 6-12 hour intervals. For each TNBS dose, 13-14 fish per group were assigned to TNBS or vehicle (30% ethanol) treatments.

**Figure 2:**
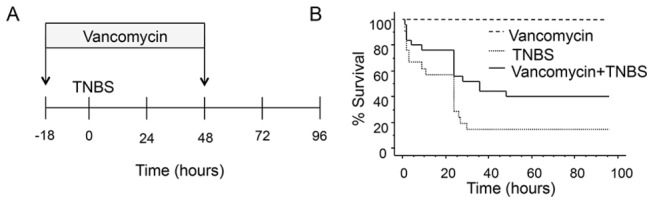
Vancomycin administration reduces mortality associated to TNBS-enterocolitis. A) Graphical representation of the study design. Vancomycin (100mg/L) was added to the fish tank water 18 hours prior to the induction of colitis. B) Kaplan-Meier analysis comparing the effect of treatment with vancomycin or vehicle on the survival of zebrafish with TNBS enterocolitis (n=21-25 fish per group).

Four different TNBS concentrations (0, 40, 80, 160 or 320 mM) were tested to find the optimal dose for subsequent experiments. Using fish survival at 96 hours post-TNBS exposure as our primary endpoint in a Kaplan-Meier analysis, we found a dose response plateau at 160 mM of TNBS (Figure 1). At this concentration, <10% of the fish survived at the end of the follow-up period. At the 24 hour time point, the number of surviving fish was 12/13 for 40 mM TNBS (p=0.2267); 8/14 for 80 mM TNBS (p<0.0004); 5/13 for 160 mM TNBS (p<0.0001); and 6/14 for 320 mM TNBS (p<0.0001, by chi-square). Under similar experimental conditions, when vancomycin (100mg/L) was added to the tank water prior to the induction of TNBS-colitis (Figure 2A), fish survival was substantially improved (50% vs 20%, vancomycin vs vehicle, n=21-25 fish per group, p=0.026 by log-rank test) (Figure 2B). 

### Histological features of TNBS-induced enterocolitis in zebrafish

**Figure 3:**
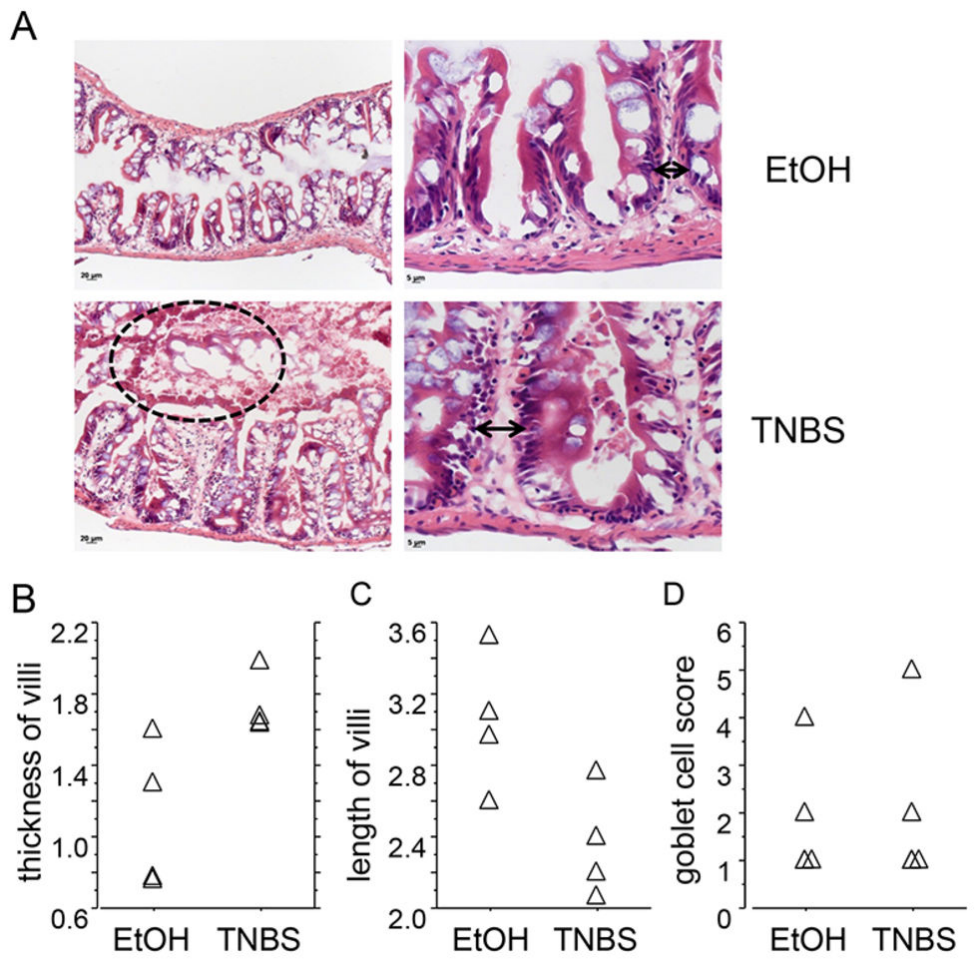
Histological features of TNBS-induced enterocolitis in adult zebrafish. A) Representative H&E stained sections form TNBS- or vehicle (30% ethanol) treated animals, at two different magnifications. Arrowheads indicate villi thickness in TNBS- and vehicle-treated intestinal sections. A region showing luminal sloughing of cellular debris in the TNBS-treated intestine is marked by a dotted line. B-C). Relative villi thickness and height (in mm) were evaluated as described in Methods. D) Abundance of goblet cells based on a 0-5 scoring system.

**Figure 4:**
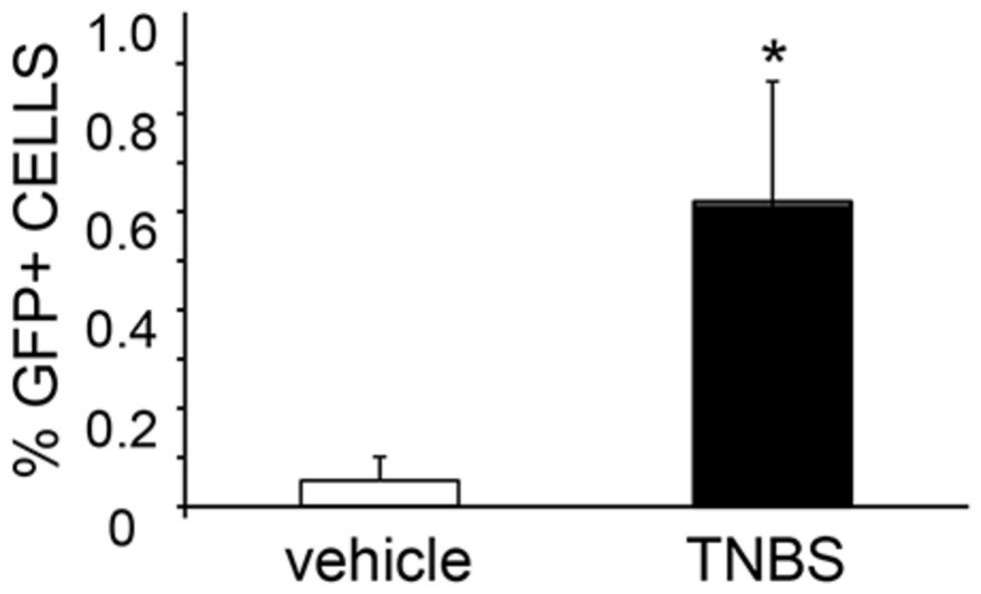
Neutrophil influx in the zebrafish intestine in response to TNBS exposure. FACS analysis of the percentage of GFP-myeloperoxidase-positive cells isolated form intestine of tg(mpx:eGFP) zebrafish 6 hours after intrarectal infusion of TNBS or vehicle (30% ethanol) (n=6-7 fish per group).

The adult zebrafish intestine is a folded tube with a single layer of epithelium that rests upon a connective tissue layer similar to the laminar propria and surrounded by circular and longitudinal muscle layers. In the epithelium, columnar-shaped absorptive enterocytes are the most numerous cells, followed by goblet cells [22]. Histological analysis of samples collected at 6 hours post-TNBS treatment revealed occasional disruption of the epithelial integrity in the form of ulcerations and sloughing of villi (Figure 3A), a characteristic previously associated with intestinal injury induced by the NSAID glafenine and an indication of improper organelle stress response leading to cellular apoptosis [28]. Similar to the oxazolone-induced model[18], we observed significant swelling of the villi (Figure 3) in fish treated with TNBS. Specifically, compared to animals treated with vehicle (EtOH), villi from TNBS-treated zebrafish were thicker (1.73±0.83 vs. 1.11±0.21, TNBS vs EtOH, p=0.021; Figure 3B) and shorter (2.36±0.15 vs. 3.05±0.19, TNBS vs EtOH, p=0.043; Figure 3C). Depletion of goblet cells, as has been described in oxazolone-induced enterocolitis[18], was not evident in our model (goblet cell abundance score 2.25±0.95 vs. 2.0±0.71, TNBS vs. EtOH, p=0.885; Figure 3D). To evaluate the extent of intestinal inflammation, infiltrating immune cells were purified from whole intestine of tg(mpx:eGFP) zebrafish treated with TNBS and subjected to FACS analysis. We found a ten-fold increase in the percentage of GFP-positive cells isolated from the intestine of zebrafish with enterocolitis: 0.614% ± 0.202% vs. 0.052% ± 0.014%, TNBS vs. vehicle treated groups (p=0.0269; n=6-7 fish per group; Figure 4), suggesting significant accumulation of neutrophils in zebrafish with acute enterocolitis.

### TNBS-treatment induced cytokine expression in the zebrafish intestine

**Figure 5:**
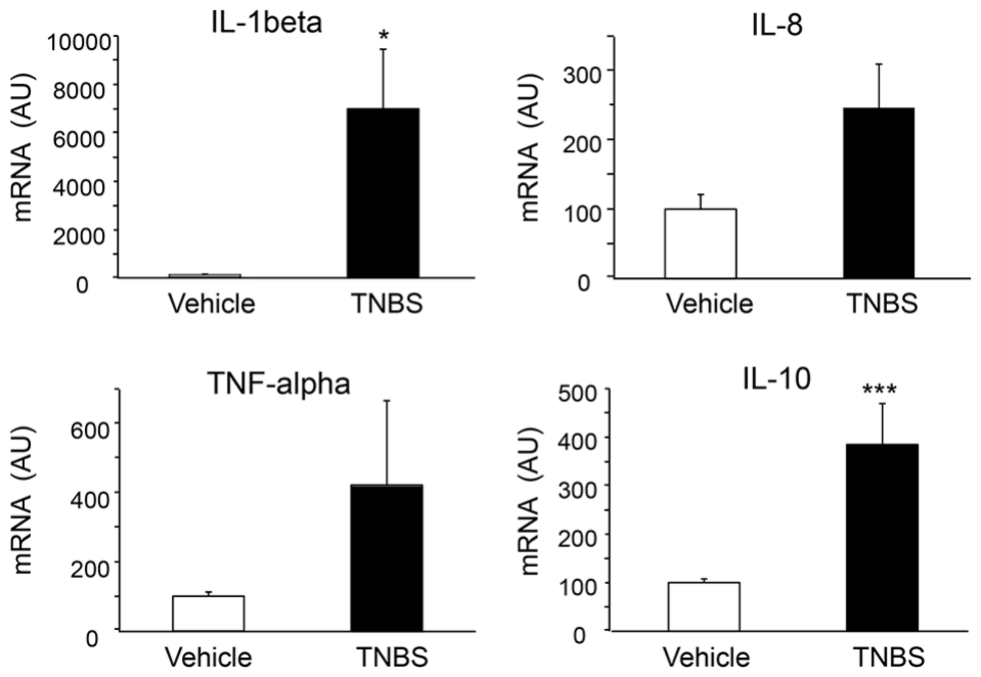
Relative mRNA expression of various cytokines in the intestine of zebrafish with TNBS-induced enterocolitis, at 6 hours post-exposure (n=9-10 fish per group). For each gene, expression in the vehicle treated group was set to 100. AU=arbitrary units.

To determine which specific cytokines respond to TNBS-induced injury in the zebrafish intestine, we measured mRNA expression of the pro-inflammatory cytokines, *IL-1beta*, *IL-8*, *TNFalpha* and the anti-inflammatory cytokine *IL-10*, as has been reported in mice with TNBS-induced colitis [12] and in zebrafish with chemical enterocolitis [14,18]. We found increased mRNA expression of *IL-1beta* (100 ± 28 arbitrary units (AU) for vehicle vs. 6,935 ± 2,454 AU for TNBS-treated; p=0.0179; n=9-10 fish per group), IL-8 (100 ± 21 AU for vehicle-treated vs. 246 ± 63 AU for TNBS-treated; p=0.0500) and IL-10 (100 ± 8 AU for vehicle-treated vs. 385 ± 84 AU for TNBS treated; p=0.0055;) following TNBS administration (Figure 5). Additionally, *TNFalpha* levels in the gut tended to be higher in the TNBS-treated fish, even though this difference did not reach significance (100 ± 12 AU for vehicle treated vs. 421 ± 243 AU for TNBS-treated; p=0.2282). These results indicate that TNBS results in an inflammatory response in zebrafish via strong induction of both proinflammatory and anti-inflammatory mediators. 

### Intestinal expression and regulation of MCH in adult zebrafish

**Figure 6:**
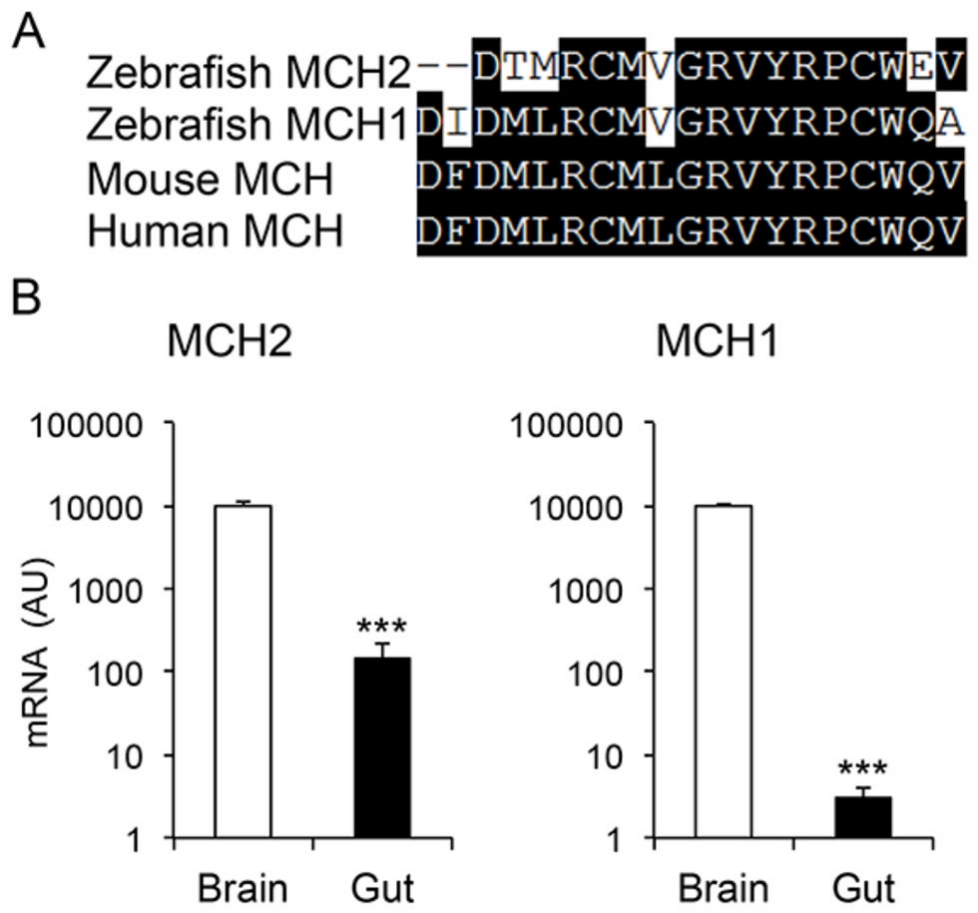
MCH expression in the adult zebrafish intestine. A) Amino acid sequence alignments of the MCH peptides from human, mouse and zebrafish. Identical residues are highlighted. B) Comparative mRNA expression of the zebrafish MCH peptides in brain and the gastrointestinal tube, presented in logarithmic scale. (n=8-9 fish per group).

The degree of homology at the protein level between human, mouse and zebrafish MCH is presented in Figure 6A. Notably, based on sequence similarity, zebrafish MCH2 appears to be more like human and mouse MCH, which are identical. Indeed, a previous report has argued that MCH2 is the human MCH ortholog [7]. Studies so far, have focused on MCH1 and MCH2 expression in the zebrafish brain. However, it has been shown in mammals that MCH is also expressed in peripheral tissues, including the immune system and the gut, though at relatively low levels under baseline conditions [13,29]. We compared MCH2 mRNA levels between the zebrafish brain and intestine, and indeed we found some expression in the latter: 10000 ± 1500 AU in the brain vs. 150 ± 70 AU in the gut (n= 8-9 per group, p<0.0001) (Figure 6B, left panel). Intestinal expression of MCH1, which has more similarity to the salmon MCH (Figure 6A), was 50- fold lower than MCH2: 10000 ± 70 AU vs. 3 ± 1.6 AU, brain vs. gut respectively, p<0.0001 (Figure 6B, right panel). 

**Figure 7:**
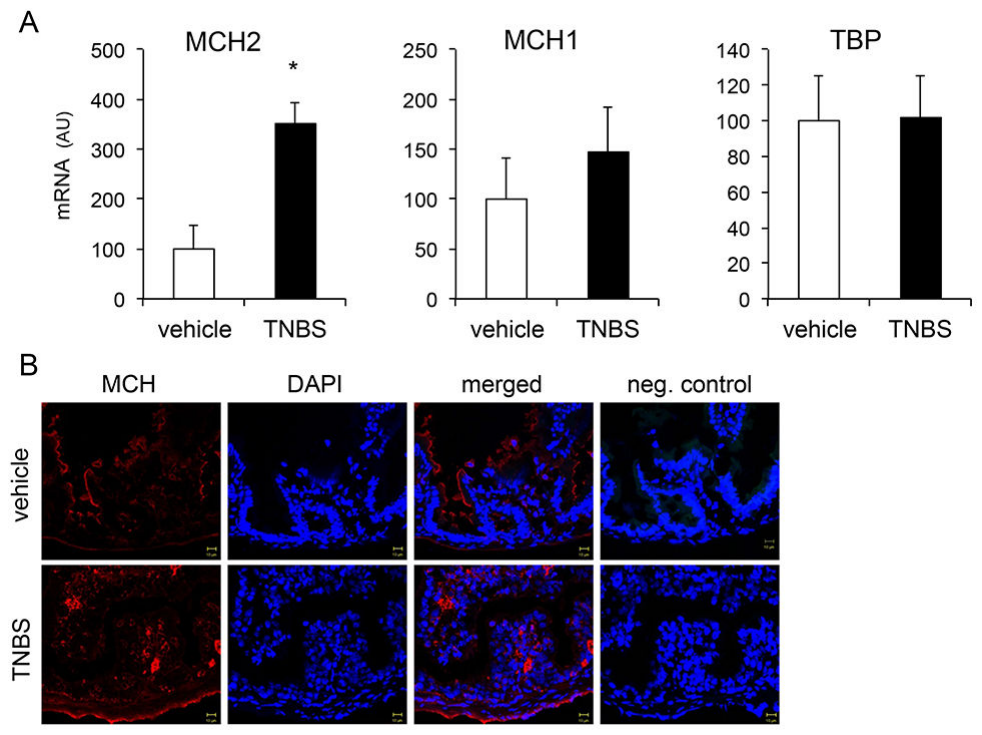
MCH intestinal upregulation in TNBS-induced enterocolitis in adult zebrafish. A) Relative mRNA expression of the two different MCH peptides MCH1 and MCH2 in the zebrafish intestine following 6 hours of TNBS intrarectal infusion. TBP serves as the housekeeping gene. (n=9-10 fish per group). For each gene, expression in the vehicle treated group has been set to 100. AU: arbitrary mRNA units. B) Immunofluorescence analysis of MCH positive cells in the zebrafish intestine at baseline and in response to TNBS treatment. An antibody against human MCH was used for staining zebrafish MCH.

Consistent with a conserved role of MCH in colitis, we found that intestinal MCH2 mRNA was significantly upregulated in zebrafish treated intrarectally with TNBS: 100 ± 41 vs. 353 ± 49 AU; vehicle vs. TNBS; n=9-10 fish per group; p=0.0065 (Figure 7A). In contrast, no changes in MCH1 expression were found following TNBS treatment: 100 ± 41 vs.148 ± 41 AU; vehicle vs. TNBS, p=0.5(Figure 7A, middle panel). Immunohistochemical analysis revealed an increase in the number of MCH-immunoreactive cells in the zebrafish intestine at 6 hours post-TNBS treatment (Figure 7B). These MCH-positive cells have not been characterized in detail in the present study, but based on morphological criteria they appear to be mononuclear phagocytes (monocytes/macrophages or dendritic cells) [30]. Although we used an antibody against human/mouse MCH in this experiment, only MCH2 mRNA was found to be upregulated in zebrafish with colitis (Figure 7, left panel), thus the MCH-staining cells most likely represent MCH2-expressing cells. 

### MCH receptor expression and regulation in the adult zebrafish intestine

**Figure 8:**
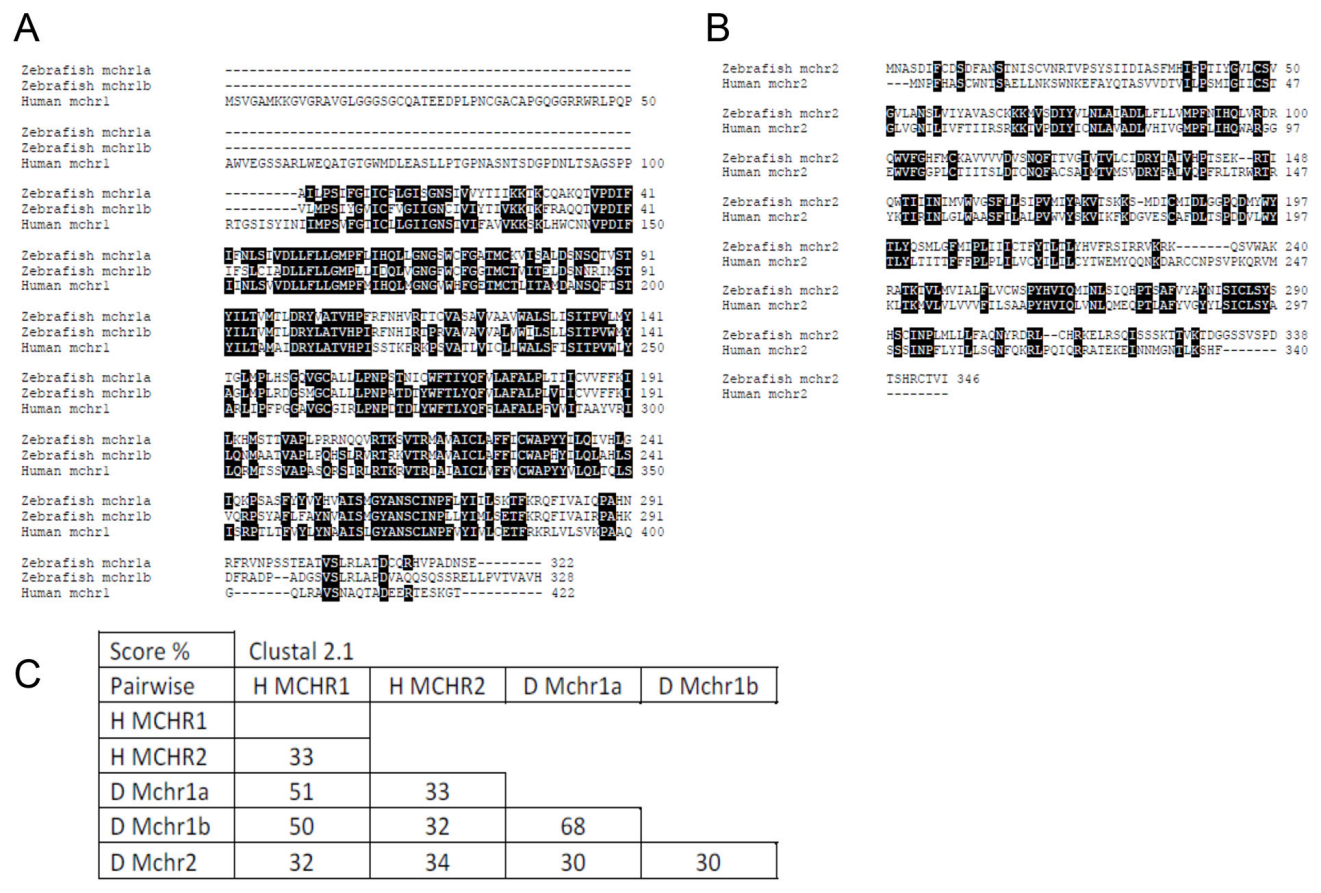
Sequence alignment of MCH receptors in various species. A) Sequence alignment of zebrafish MCHR1a, MCHR1b and human and mouse MCHR1. B) Sequence alignment of MCHR2 receptors in zebrafish, human and mouse . Identical residues are highlighted. C) Graphical representation of sequence identity for MCH receptors among species.

Since both MCH and its receptors were found to be upregulated in the intestine of patients with inflammatory bowel disease and of mice with TNBS-induced colitis [12], we subsequently examined mRNA levels of MCHR1b and MCHR2 in brain vs. gut and in response to TNBS treatment. As shown in Figure 8A, at the protein level the two isoforms of zebrafish MCHR1, i.e. MCHR1a and MCHR1b, share about 50% homology to their human equivalent. Zebrafish MCHR2 has about 30% homology with either one of the human receptors and with zebrafish MCHR1 (Figure 8B-C). 

**Figure 9:**
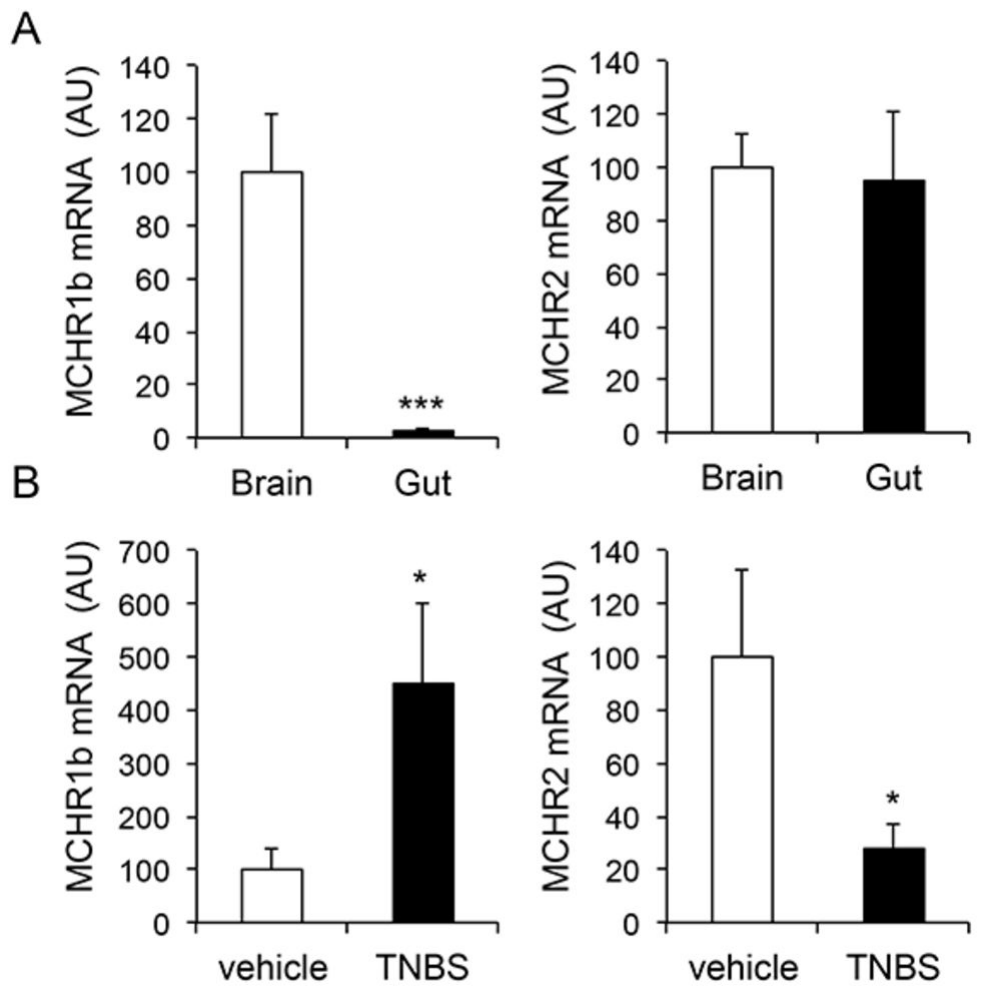
Zebrafish MCH receptor mRNA expression and regulation by intestinal inflammation. A) Relative expression of zebrafish MCHR1b and MCHR2 in brain and the intestine. B) Expression of intestinal MCHR1b and MCHR2 mRNA following treatment with TNBS or vehicle (n=9-10 fish per group). Values in the vehicle treatment group are set to 100. AU: arbitrary units.

Two previous studies report zebrafish MCHR1a and MCHR1b mRNA expression in the zebrafish brain (MCHR1a only transiently during development) while MCHR2 expression was more prominent in the skin [7,10]. In our analysis, MCHR1b expression in the gut was only 2% of that in brain tissue (100 ± 22 in the brain vs. 2.5 ± 1.2 in the gut; n=8-9 fish per group; p=0.0009) (Figure 9A). We also found that MCHR1b was upregulated in the zebrafish intestine in response to TNBS treatment: 100 ± 40 vs. 452 ± 151 AU, vehicle vs. TNBS; p=0.0469; n=9-10 fish per group (Figure 9B, left panel). Surprisingly, MCHR2 was found to be downregulated in the intestine of TNBS-treated fish (100 ± 34 vs. 28 ± 9; vehicle vs. TNBS; p=0.0456 (Figure 6B, right panel). Taking into consideration that increased expression of human MCHR2 was found during intestinal inflammation [12], this observation might suggest differences in acute vs. chronic colitis, or perhaps that zebrafish MCHR1b may exhibit more functional homology to human MCHR2 in the zebrafish intestine. 

## Discussion

In the present study we describe a model of chemically-induced enterocolitis in adult zebrafish that recapitulates several aspects of the human and mouse disease. In parallel, we demonstrate the presence of MCH and its receptors in the zebrafish intestine, and their regulation by inflammation, suggesting that certain functions of MCH are conserved across species. Murine models have been extremely useful in the study of the pathogenesis of many diseases, including inflammatory bowel disease (IBD), and for testing potential therapies [31-33]. However, experiments in mice are limited by the time, cost, and ethical constraints of generating and characterizing multiple transgenic lines. Zebrafish have a relatively rapid life-cycle and low maintenance expenses, are easy to breed and highly amenable to genetic manipulation. For these reasons, zebrafish make an attractive model for studying inflammatory bowel disease. An increasing number of similarities have been described in both innate and adaptive immune responses and anatomical and functional conservation has been reported between zebrafish and mammalian intestinal structure and function [17,22,23]. 

 Induction of colitis by intrarectal administration of the hapten TNBS has been largely used in rodent studies and recently it has been introduced in zebrafish larvae [14]. In mammalian experiments, TNBS causes a strong inflammatory response within hours of exposure by modulating endogenous and bacterial antigens. This inflammatory response is characterized by severe destruction of the epithelium and massive influx of immune cells [34]. However, we have previously reported that MCH-deficient mice develop attenuated inflammation in response to TNBS [12]. This was also reflected in lower colonic TNFalpha and IL-1beta levels compared to wild type mice at 48h post treatment. In the present study, TNFalpha and IL-1beta were induced in the adult zebrafish model 6 hours after intrarectal instillation of TNBS. Furthermore, as in the mouse studies, we detected significant accumulation of myeloperoxidase-positive cells in the zebrafish intestine (Figure 4). 

The only previous model of adult zebrafish enterocolitis employed intrarectal administration of oxazolone[18]. Under the conditions tested, we did not observe any striking differences between these two models in their acute phase, which is characterized by granulocyte influx in response to acute intestinal injury. Another important similarity of zebrafish chemical enterocolitis induced by TNBS or oxazolone is the requirement of an intact microbiome for full disease development [16,18]. The significance of the microbiome in the IBD pathogenesis has been recently underscored by clinical improvement of patients receiving fecal transplants from healthy donors [35,36]. Whether in their chronic phase, TNBS- and oxazolone-elicited immune responses differ in zebrafish as in the mouse experimental colitis remains to be seen. For instance, in mice, TNBS causes an IL-12 mediated Th1 type of immune response resulting in a transmural inflammation resembling Crohn’s disease. Oxazolone, on the other hand, has been associated with IL-4 and IL-13 production and a Th2 immune response reminiscent of ulcerative colitis [34]. 

In relation to MCH, we have found several-fold upregulation of MCH and MCHR1 mRNA expression in the colon of mice with TNBS colitis (unpublished data) and in patients with inflammatory bowel disease [12], as we report here for their zebrafish orthologs, MCH2 and MCHR1b, respectively. While humans and zebrafish possess a second receptor for MCH, MCHR2, this is not the case in rodents. Thus, in theory, zebrafish might represent a suitable model to study the role of MCHR2 in colitis, particularly in light of its intestinal regulation that we demonstrated in TNBS-exposed zebrafish. The fact that MCHR2 was found to be upregulated in patients with chronic IBD [12], but downregulated in zebrafish, might simply indicate a difference in acute vs. chronic inflammation; nevertheless MCHR2’s role in intestinal inflammation, in particular if opposite of that of MCHR1, warrants further investigation in the zebrafish model.

 This first report of MCH expression in the zebrafish intestine, demonstrated by both RT-PCR and immunostaining, fits well with the evolving concept of evolutionarily conserved neuropeptides of the brain-gut axis modulating inflammatory responses [37]. For instance, orthologs for CRH and urocortins [38], Substance P [39], NPY [39], alpha-MSH [40], ghrelin [41] and GIP [42] have been described in zebrafish and in parallel implicated in the pathogenesis of intestinal inflammation, in mouse and human studies [43-49]. It appears that these molecules are highly conserved in evolution, not only in sequence but also from a functional perspective, and have essential roles in the regulation of food intake, stress response and defense mechanisms, all critical elements for advancement of the organism’s survival. Thus, the TNBS-induced model of enterocolitis in adult zebrafish established in this report might be used as a tool to expand our knowledge on neuropeptide pathophysiology. In parallel, it will allow for the identification of previously unappreciated pathways of importance to the pathogenesis of IBD and effective screening of new drug candidates, including agents targeting MCH, or repurposing of old drugs as has been recently suggested for a mu-opioid agonist based on its effects on a zebrafish model of intestinal injury [28].

## Supporting Information

Table S1 **Quantitative RT-PCR primer sequences with citations.**
(DOCX)
