# Supplementary material for: Intestinal Upregulation of Melanin-Concentrating Hormone in TNBS-Induced Enterocolitis in Adult Zebrafish
Source: PLoS One. 2013 Dec 20;8(12):e83194. doi: 10.1371/journal.pone.0083194 (PMC3869761; doi:10.1371/journal.pone.0083194)
Supplement: Table S1 — Quantitative RT-PCR primer sequences with citations. (DOCX) [file pone.0083194.s001.docx]

**Supplementary Table 1: Quantitative RT-PCR primer sequences with citations**

| **Gene name** | **Forward primer** | **Reverse primer** | **Accession number** | **Ref. #** |
| --- | --- | --- | --- | --- |
| TBP | 5’-ACCACTGCTCTGTTGTTTTGC-3’ | 5’-TTCTGGGTTCTCGTATTCTCAT-3’ | AF503449.1 | 24 |
| IL-1β | 5’-TGCGGGCAATATGAAGTCA-3’ | 5’-TTCGCCATGAGCATGTCC-3’ | NM_212844 | 18 |
| IL-8 | 5’-TGTGTTATTGTTTTCCTGGCATTTC-3’ | 5’-GCGACAGCGTGGATCTACAG-3’ | XM_001342570.3 | 26 |
| TNF-α | 5’-AGACCTTAGACTGGAGAGATGAC-3’ | 5’-CAAAGACACCTGGCTGTAGAC-3’ | BC165066.1 | 26 |
| IL-10 | 5’-AGGGCTTTCCTTTAAGA-3’ | 5’-ATATCCCGCTTGAGTTCC-3’ | BC163031.1 | 18 |
| Pmch2 | 5’-TGCGGACACAGGAATTAAAGG-3’ | 5’-ATCCATCGTGCTGAATCCATC-3’ | NM_001202542.1 | 25 |
| Pmch1 | 5’-ATCATCGTGGTGGCTGACTCC-3’ | 5’-GCTTTCGGGTGCGTTGAGATG-3’ | FJ204827.1 | 25 |
| MCHR1a | 5’-AAATGCCAGGCTAAACAAACA-3’ | 5’-AAGACGAAGGGACACAGTGG-3’ | AY161857.1 | 10 |
| MCHR1b | 5’-TGGTGTGGATCCTCTCACTG-3’ | 5’-CCGGATGGCAACAATAAACT-3’ | AY161858.1 | 10 |
| MCHR2 | 5’-TTGCAATCGTCCATCCTACA-3’ | 5’-CTGGTGGGATGCTGGATACT-3’ | AY161859.1 | 10 |
